# Associations between subjective cognitive concern, brain network connectivity, and cognitive performance in cognitively normal older adults

**DOI:** 10.1016/j.nbas.2025.100155

**Published:** 2025-12-12

**Authors:** Kimberly Albert, Brian Boyd, Brenna McDonald, Julie Dumas, Andrew Saykin, Warren Taylor, Paul Newhouse

**Affiliations:** a The Center for Cognitive Medicine, Department of Psychiatry and Behavioral Sciences, Vanderbilt University Medical Center, Nashville, TN, the United States of America; b Department of Radiology and Imaging Sciences, Indiana University School of Medicine, Indianapolis, IN, the United States of America; c Clinical Neuroscience Research Unit, Department of Psychiatry, University of Vermont College of Medicine, Burlington, VT, the United States of America; d The Geriatric Research, Education and Clinical Center, Department of Veterans Affairs Medical Center, Tennessee Valley Healthcare System, Nashville, TN, the United States of America

**Keywords:** Subjective cognitive decline, cognitive compensation, functional MRI, functional connectivity, Default mode network, Salience network, visual-spatial processing, attention

## Abstract

Subjective Cognitive Decline (SCD) is the perception of a persistent decline in cognitive function and self-reported concerns over cognitive ability in older adults with normal objective cognitive performance. SCD is associated with increased Alzheimer’s Disease (AD) risk and early AD pathology. The neurobiological underpinnings of SCD and cognitive or neural circuit alterations during SCD remain unclear. This study aimed to identify patterns of brain network functional connectivity that are associated with quantitative measures of cognitive concerns, and to examine how these functional patterns are related to performance in the cognitive domains of visual-spatial processing, attentional control, and working memory. This analysis combined data from three studies of cognitively healthy older adults which included a quantified assessment of cognitive concern severity, resting-state fMRI, and cognitive testing in the above domains. We examined brain network-to-network functional connectivity associated with self-rated cognitive concern severity, and then how the identified patterns relate to cognitive performance. Results showed that greater cognitive concern severity was associated with unique patterns of functional connectivity between the Default Mode Network and the Language and Salience Networks in older adults without objective cognitive impairment. While greater cognitive concern severity alone was associated with slower processing reaction time, these functional connectivity patterns were associated with faster processing reaction time. This suggests that these functional connectivity patterns may alter the relationship between cognitive concern severity and psychomotor slowing. These findings support that despite the perception of cognitive changes in older adults, normal cognitive performance may be maintained through functional connectivity changes in brain networks important to directing visual-spatial attention and processing.

## 1 Introduction

Subjective Cognitive Decline (SCD) is the perception of a persistent decline in cognitive function and concern over cognitive performance in older adults with normal objective performance in clinical cognitive testing [1,2]. SCD is associated with increased risk for progression to mild cognitive impairment (MCI) and Alzheimer’s Disease (AD) dementia beyond the effects of age, education, and genetic risk factors [[1], [2], [3], [4], [5]]. The risk of progression to AD is two times greater in those with SCD than without [5]. Rates of AD neuropathology are increased in SCD[6], [7], [8], [9], [10], [11], [12], as are structural and functional brain changes similar to those observed in MCI and AD [13].(i.e., cortical thinning, temporal lobe atrophy[14], [15], [16], [17], [18], hypometabolism and altered functional connectivity [13]). It is possible that in the early stages of SCD, brain network alterations may help to maintain objectively measured performance. These changes may be experienced as increased effort to engage in cognitive tasks, resulting in the perception of cognitive decline despite normal objective performance and result in cognitive concerns or complaints.

SCD may reflect an early stage of cognitive change before neuropathology becomes widespread enough to impair objective performance and meet diagnostic criteria for MCI [19], [20]. After the onset of significant impairment, AD pathology may be progressed to the point that treatment strategies aimed at modifying disease progression and maintaining cognitive function is less effective[21]. Targeting the earliest stages of pathological cognitive aging may provide a better opportunity for interventions aimed at maintaining cognitive function[22].

There are proposed research criteria defining SCD[1] using component features (including subjective cognitive concern, age and recency of change, informant confirmation, genotype, and AD biomarker status) that increase the likelihood that an individual is experiencing subjective cognitive decline in relation to preclinical AD. However, SCD is not currently a clinical diagnosis and there is no standardized assessment or quantitative thresholds for subjective cognitive concern severity that define SCD[1]. While many studies have investigated SCD using a binarized categorization (often based on endorsing concern over memory in aging or seeking memory support without a MCI/AD diagnosis), our [23] previous work demonstrates that cognitively normal older adults experience a wide range of cognitive concerns [23], [24], [25], [26]. The severity of subjective cognitive concerns with aging may reflect different levels of subtle functional decline or cognitive compensation required to maintain normal cognition. This study examines the relationship between a quantitative measure of subjective cognitive concerns, brain network functional connectivity, and cognitive performance in older adults, Understanding the neurobiological mechanisms and network changes associated with subjective cognitive concerns and potential cognitive compensation may help identify early cognitive changes in SCD and novel targets for cognitive intervention.

In early AD pathological changes in temporal regions begin to impact memory function, resulting in subtle memory decline [27]. Compensation models of cognitive aging posit that in response to declining memory function, alternative brain networks and cognitive systems are engaged to maintain memory performance [28], [29]. These compensatory changes may involve cognitive processes that are primary to perceptual function and cognitive control such as processing speed, visual-spatial attention, and working memory [28]. Although recruiting these processes may support subsequent long-term memory and global cognitive function[30], [30], [31], [32], [33], [34], [35], [35], [36], [37], [38], [39], [40], [41], [42], the greater reliance on these mechanisms may be experienced as increased effort, contributing to the perception of subjective decline and great cognitive concern severity [43], [44], [45].

In addition to brain structural change similarities between SCD and MCI/AD[7], [16], [17], [18], [46], [47], [48], [49], [50], [51], brain network activity and functional connectivity changes have been associated with SCD and subjective cognitive concern severity. Changes associated with SCD include [53] increased task-based activity and functional connectivity of the fronto-parietal cognitive control network and Default Mode Network (DMN) [46], [52]. SCD has been associated with increased task-related activity during memory processing in dorsolateral prefrontal cortex [53], [54]. Additionally, subjective cognitive concern severity is associated with increased metabolism in insula, inferior parietal, lingual, and fusiform regions[55], [56].[55], [56]. Increased regional activity and network functional connectivity is consistent with hyperconnectivity seen in preclinical AD before objective decline and during mild decline. Preclinical AD and early amyloid accumulation have been associated with brain network hyperconnectivity, particularly between DMN and Salience network (temporal and parietal) regions [57], [58], [59].

However, brain activity findings in SCD have been inconsistent including decreased memory-related activity in hippocampal, superior parietal, occipital and posterior cingulate regions [53], [54], [60] and decreased DMN functional connectivity[46], [61], [62], [63]. These heterogenous findings may result from inconsistencies in the methods used to define SCD and the limited inclusion of quantified subjective cognitive concern severity. Additionally, few studies have examined the relationship between functional connectivity differences associated with subjective cognitive concerns in aging and cognitive performance which would help distinguish between the effects of subtle decline versus compensatory changes. [46], [52], [55].

The goal of this study was thus to identify brain network functional connectivity patterns that are associated with greater self-reported cognitive concern in older adults who have not been diagnosed with MCI or dementia. We also examined the relationship between these connectivity patterns and performance in the primary cognitive domains of visual-spatial processing, attentional control, and working memory. Our hypothesis was that greater cognitive concern severity would be associated with stronger negative functional connectivity between the DMN and networks involved in task-oriented processing of external stimuli (Visual, Salience, Dorsal Attention), in accordance with DMN hyperconnectivity often seen in SCD and preclinical AD. We secondarily hypothesized that these functional connectivity patterns would be associated with better performance in visual-spatial processing, attentional control, and working memory. These findings would provide evidence of compensatory brain network functional changes that support cognitive performance in individuals with cognitive concerns and/or SCD. Understanding the mechanisms that support normal cognitive performance in such individuals may help determine the neurobiological basis of the SCD phenomenon. Further, these findings could identify characteristics of brain activity that, in further longitudinal studies, reflect enhanced dementia risk and/or possible targets for future interventions to support cognitive function in early AD.

## 2 Methods

Data were collected from three studies of healthy, cognitively normal older adults who had completed Cognitive Complaint Index (CCI) [64] scores, resting-state fMRI, and cognitive testing including the Choice Reaction Time (CRT) [65] task, Posner task [66], and letter N-Back task [67] 0-, 1-, 2-, and 3-back conditions.

The studies included the Cognitive Concern (CC) study, the Aging, COgnition, and Brain Activity (ACOBA) study, and the Changes in Attention Network Dynamics in Aging (Changes) study. These studies were included because they had similar entry criteria for participants, were completed on the same MRI scanner hardware models with similar resting-state fMRI scanner sequences, and used the same testing programs and protocols for the cognitive tasks.

### 2.1 Participants

These studies were approved by either the Vanderbilt University or University of Vermont Institutional Review Board and informed consent was obtained from all participants. Participants were recruited through posted notices and advertisements in local newspapers, email listservs, and direct mailings.

Participants were older adults without 1) current or past psychiatric, neurological, or neurocognitive disorders; 2) significant systemic illness or unstable medical condition; 3) dementia or cognitive impairment (MMSE [68] or MoCA [69] > 26). Participants were not taking psychotropic medications and women had not taken ovarian hormone therapy within the last 12 months. Participants were further screened for normal cognition and the absence of MCI/AD using the Older Adult Self Report [70] (memory and cognition problems within the normal range for age and sex), and Brief Cognitive Rating Scale (score ≤ 2) [71] to establish a Global Deterioration Scale score (GDS; score ≤ 2) [72].

### 2.2 Cognitive concern severity

Subjective cognitive concern severity was operationalized as the percent of items endorsed on the self-reported Cognitive Complaint Index (CCI) [23], [24], [25], [26], [64], [73], [74]. The CCI is a 120-item questionnaire that provides quantifiable measures of subjective perception of changes in memory, learning, and activities of daily living, and concerns about these perceived changes. [64]. Additionally, the CCI queries change over time, thus assessing a pattern of change from past performance rather than only acute concerns [2], [64], [75]. Each item was binarized to represent a yes or no endorsement of the item. As 6 items of the CCI are short-answer responses, only the remaining 114 items were included in the binarized CCI score (percent of CCI items endorsed). CCI score was used as a continuous measure for all analyses.

The association between CCI score and cognitive performance was analyzed using multiple regression controlling for age, sex, and education in R (4.3.2).

### 2.3 Cognitive performance

Cognitive performance was assessed for the domains of visual-spatial processing, attentional orienting and shifting, and working memory. These domains represent primary cognitive processes involved in memory and learning [76], [77], [78] and were assessed in each of the parent studies.

Visual-spatial processing was assessed as psychomotor reaction time (RT) using the Choice Reaction Time task (CRT) [65]. During the CRT participants must hold a central “home” button down until a light appears above one of several other buttons, then move to push that button as quickly as possible. This task distinguishes between processing RT and motor RT. Processing RT is measured as the time between the light appearing and the participant releasing the “home” button. Motor RT is measured as the time between the participant releasing the “home: button and pressing the button corresponding to the light. Median motor RT, processing RT, and total RT were used as outcome measures. Total RT is included as comparable to other reaction time tasks that do not separate processing and motor RT.

Attentional orienting and shifting were assessed using the Posner Task [66] which assesses the ability of participants to disengage attention and shift to a new target. Participants are presented targets on the right and left side and must quickly indicate which side the target appears. Trials are preceded by no cue, spatially neutral cues, spatially valid, and spatially invalid cues (directing attention away from the side where the target will appear). Median RTs for validly and invalidly cued targets were used as outcome measures for attentional orienting (valid-no Cue RT, reflecting the ability to use a cue to orient visual-spatial attention) and shifting (valid-invalid RT, reflecting the ability to shift visual-spatial attention from a previously attended location to a new location) respectively.

Working Memory was assessed using a letter N-Back Task [79] in which participants are presented a sequence of letters and must indicate if the current letter is the same as the one presented N trials previously (0-, 1-, 2-, 3-back). d’ is a measure of signal detection sensitivity and was used as a measure of task accuracy. The difference in d’ for 2- vs. 0- and 3- vs. 0-back conditions was used as the outcome measure.

### 2.4 MRI acquisition

Participants completed one MRI scan session that included structural MRI and functional (fMRI) during resting-state. Participants also completed cognitive tasks during the MRI scan sessions that differed by study and are not included in these analyses nor reported here.

At the University of Vermont and Vanderbilt, imaging data were collected using identical 3 T Philips Achieva MRI (Philips Medical Systems, Inc., Best, Netherlands) scanners. Both scanners were identical in software and hardware. Resting state scans were completed in all studies at both sites using the same resting-state sequence and with participants instructed to maintain eyes open and looking at a focus cross on the screen in the scanner. All data were processed together at Vanderbilt. Scanner site has been included in the model for prior analyses using the imaging data collected at both sites and no significant effect was observed (Albert et al., 2015). Resting-state fMRI was collected in the absence of external stimuli using an fMRI resting SENSE sequence (FOV = 240 mm2, matrix size = 80 × 80, 3 × 3 × 5 mm3 voxels, TR = 1500 ms, TE = 35 ms, flip angle = 90°, 0 mm gap, 5 mm slice thickness, 24 axial slices, 256 volumes). A high-resolution T1-weighted (T1W) fast field echo structural scan (FOV = 256 mm2, 1 mm isotropic voxels, TR = 9.8 ms, TE = 4.6 ms, flip angle = 8°, 140 sagittal slices) was collected to provide a template for image registration.

### 2.5 Functional connectivity

Results included in this manuscript come from analyses performed using CONN [80] (RRID:SCR_009550) release 21.a [81] and SPM [82] (RRID:SCR_007037) release 12.7771.

*Preprocessing:* Functional and anatomical data were preprocessed using a flexible preprocessing pipeline [83] including realignment with correction of susceptibility distortion interactions, slice timing correction, outlier detection, direct segmentation and MNI-space normalization, and smoothing. Functional data were realigned using SPM’s realign & unwarp procedure [84], where all scans were coregistered to a reference image (first scan of the first session) using a least squares approach and a 6-parameter (rigid body) transformation [85] and resampled using b-spline interpolation to correct for motion and magnetic susceptibility interactions. Temporal misalignment between different slices of the functional data (acquired in interleaved Philips order) was corrected following SPM’s slice-timing correction (STC) procedure [86], [87], using sinc temporal interpolation to resample each slice’s BOLD timeseries to a common mid-acquisition time. Potential outlier scans were identified using ART as acquisitions with framewise displacement above 0.9 mm or global BOLD signal changes above 5 standard deviations [88], [89], and a reference BOLD image was computed for each subject by averaging all scans excluding outliers. Functional and anatomical data were normalized into standard MNI space, segmented into grey matter, white matter, and CSF tissue classes, and resampled to 2 mm isotropic voxels following a direct normalization procedure [89], [90] using SPM’s unified segmentation and normalization algorithm [91], [92] with the default IXI-549 tissue probability map template. Last, functional data were smoothed using spatial convolution with a Gaussian kernel of 6 mm full width at half maximum (FWHM).

*Denoising:* Functional data were denoised using a standard denoising pipeline [93] including the regression of potential confounding effects characterized by white matter timeseries (5 CompCor noise components), CSF timeseries (5 CompCor noise components), motion parameters and their first order derivatives (12 factors) [94], outlier scans (below 38 factors) [88], session effects and their first order derivatives (2 factors), and linear trends (2 factors) within each functional run, followed by bandpass frequency filtering of the BOLD timeseries [95] between 0.008 Hz and 0.09 Hz. CompCor [96], [97] noise components within white matter and CSF were estimated by computing the average BOLD signal as well as the largest principal components orthogonal to the BOLD average, motion parameters, and outlier scans within each subject's eroded segmentation masks. From the number of noise terms included in this denoising strategy, the effective degrees of freedom of the BOLD signal after denoising were estimated to range from 49.7 to 87.6 (average 69) across all subjects [89].

*First-level analysis***:** ROI-to-ROI connectivity matrices (RRC) were estimated characterizing the patterns of functional connectivity with 184 ROIs within the CONN network atlas. Functional connectivity strength was represented by Fisher-transformed bivariate correlation coefficients from a weighted general linear model (weighted-GLM [98]), defined separately for each pair of ROIs, modeling the association between their BOLD signal timeseries. In order to compensate for possible transient magnetization effects at the beginning of each run, individual scans were weighted by a step function convolved with an SPM canonical hemodynamic response function and rectified.

*Second-level analysis – Effect of CCI Score:* The relationship between cognitive concern severity and resting-state functional connectivity was analyzed using multiple regression with CCI score as the independent variable, controlling for the effect of age, sex, education, and study. Second-Level ROI-to-ROI connectivity matrices were estimated for CONN network atlas ROIs for: Default Mode, Visual, Sensorimotor, Fronto-Parietal, Salience, Language, and Dorsal Attention.

All CONN atlas ROIs (184) for each network were included and analyses were conducted for between network functional connectivity. 2nd-level analyses were performed using a General Linear Model (GLM [99]). Results were thresholded using a combination of a cluster-forming p < 0.001 voxel-level threshold to identify significant voxels and correction for multiple comparisons using contiguous voxel clustering with a familywise corrected p-FDR < 0.05 cluster-size threshold [100].

ROI clusters with functional connectivity significantly correlated with CCI score were identified, and functional connectivity strength was extracted for further analyses including association with cognitive performance. ROI clusters are groups of regions within the same network that show the same connectivity pattern (same direction of inter-network connectivity with the same region in another network).

*Functional Connectivity and Cognitive Performance*.

The relationship between the average functional connectivity strength of each identified significant between network connection and cognitive performance measures was analyzed using multiple regression controlling for age, sex, and education in R (4.3.2). Extreme outlier values (> 3 times the IQR below Q1 or above Q3) were removed from analyses. Post-hoc analyses examined the relationship between functional connectivity strength for each identified significant ROI-ROI pair and cognitive performance measures, and were not corrected for multiple comparisons.

## 3 Results

### 3.1 Participants

Participants (n = 86, Table 1) had a mean age of 63.70 years (SD = 7.93) and mean education of 16.09 years (SD = 2.49). There were no significant differences in participant age (F(1,84) = 0.04, p = 0.84) or education (F(1,84) = 0.09, p = 0.77) by study (Cognitive Concern study n = 33, ACoBA n = 20, Changes study n = 33). Participants were 69 % women; the Cognitive Concern study limited enrollment to only women, ACoBA had 40 % women, and the Changes study had 55 % women). No participants were taking psychotropic medications including antidepressants and anxiolytics. The mean CCI score was 32.0 % (SD = 16 %). Previous studies have used a threshold of 20 % item endorsement to identify SCD [23], [24], [26], [64]; 67 participants had CCI scores above the 20 % cutoff for SCD. There were significant differences in CCI score by study (F(1,84) = 7.44, p < 0.005) as only the ACOBA study required participants to have a CCI score of above 20 % endorsement. However, for the purposes of this study CCI score was used as a continuous measure of cognitive concern severity and SCD classification was not used in the analyses.

**Table 1:** Participants.

			**Data Source Study**					
	**Total (n = 86)**		**Changes (n = 33)**		**CC (n = 33)**		**ACOBA (n = 20)**	
	**Mean**	**SD**	**Mean**	**SD**	**Mean**	**SD**	**Mean**	**SD**
**Age**	63.70	8.17	67.73	7.27	55.82	3.21	69.80	4.14
**Sex** **(% Female)**	0.69 (n = 55)		0.55 (n = 18)		1.00 (n = 33)		0.40 (n = 8)	
**Education**	16.17	2.50	16.03	2.76	16.30	0.14	16.20	2.50
**CCI Score** **(% endorsed)**	0.32	0.16	0.31	0.17	0.26	0.14	0.45	0.10
**MoCa**			28.22	1.42			27.18	1.01
**MMSE**					28.93	1.25		
CC: Cognitive Concerns study, ACOBA: Aging, COgnition, and Brain Activity study, CCI: Cognitive Complaint Index, MoCA: Montreal Cognitive Assessment, MMSE: Mini Mental State Exam								

### 3.2 CCI score and cognitive performance

Extreme outliers based on cognitive task performance were identified and removed from analyses for CRT median motor RT (1 outlier score), CRT median total RT (1 outlier score), N-Back d’3-d’0 (3 outlier scores), N-Back d’2-d’0 (1 outlier score). Scores for uncompleted tasks were also omitted from analyses (Table 2).

**Table 2:** CCI score and cognitive performance.

					**CCI effect**			
	**Cognitive Measure**	**Scores Included**	**Mean**	**SD**	**df**	**F**	**p**	**β**
**CRT**	Processing RT	81	418.99	63.51	76	6.03	**0.02**	31.19
	Motor RT	80	378.01	94.91	75	0.05	0.82	
	Total RT	80	804.42	128.96	75	2.56	0.11	
**Posner**	Invalid RT	77	454.98	76.04	72	0.06	0.80	
	Valid RT	79	401.82	84.82	74	1.60	0.21	
	Alert	78	−76.67	77.09	73	0.17	0.68	
	Orient	77	−107.67	88.35	72	0.04	0.85	
	Re-Orient	77	−47.31	47.02	72	1.43	0.24	
**N-Back**	d’ 3–0	75	−1.93	0.89	70	0.70	0.41	
	d’ 2–0	77	−1.65	0.93	72	42.00	0.52	
RT: reaction time, CRT: critical reaction task								

CCI score was significantly positively associated with CRT median processing RT (Table 2), with higher CCI score associated with longer processing RT. CCI score was not significantly associated with CRT motor RT or total RT. CCI score was also not significantly associated with Posner or N-Back task performance.

### 3.3 CCI score and Network functional connectivity

**Fig. 1:**
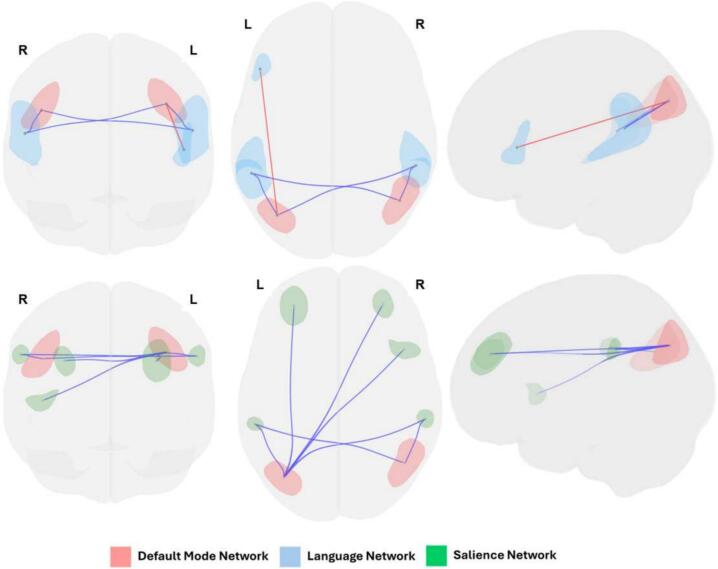
**Inter-network ROI to ROI functional connectivity that is significantly associated with Cognitive Complaint Index score.** Default Mode Network (DMN, red ROIs: bilateral lateral parietal) functional connectivity with multiple regions in the Language (blue ROIs: left inferior frontal gyrus and bilateral posterior superior temporal gyrus) and Salience (green ROIs: bilateral rostral prefrontal cortex, right anterior insula, and bilateral supramarginal gyrus) had a significant association with Cognitive Complaint Index (CCI) score. Only inter-network connections were included. Higher CCI score was associated with stronger functional connectivity between DMN and Language and Salience Network regions. The associations included both negative (blue lines) and positive (red lines) connectivity between Default Mode Network and Language Network regions, and only negative connectivity between Default Mode Network and Salience Network regions. (For interpretation of the references to colour in this figure legend, the reader is referred to the web version of this article.)

Higher CCI score was associated with ROI-to-ROI functional connectivity (Fig. 1) between the DMN lateral parietal (LP) regions bilaterally and two ROI groups in the Language and Salience Networks.

**Fig. 2:**
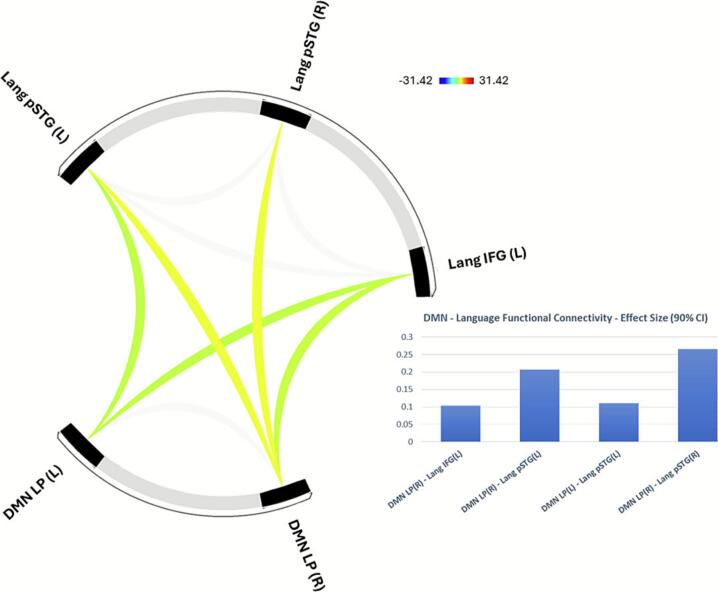
**Default Mode Network – Language Network Functional Connectivity.** Default Mode Network (DMN) lateral parietal (LP) ROIs showed positive functional connectivity with Language (Lang) Network ROIs including posterior superior temporal gyrus (pSTG) and inferior frontal gyrus (IFG).

*Functional Connectivity between DMN and Language Network*: DMN showed positive functional connectivity with Cluster 1 Language Network regions including the inferior frontal gyrus (IFG) and posterior superior temporal gyrus (pSTG) (F(3,83) = 69.86, p-corrected < 0.001, Fig. 2). In these analyses, higher CCI score was associated with higher positive functional connectivity between the DMN left LP region and left IFG. Conversely, higher CCI score was associated with lower positive functional connectivity between DMN bilateral LP regions and pSTG (F(3,77) = 7.63, p-corrected < 0.01, Fig. 4).

**Fig. 3:**
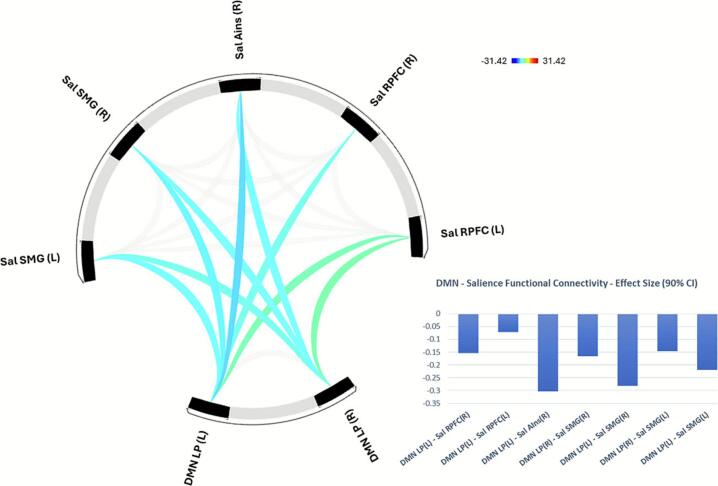
**Default Mode Network – Salience Network Functional Connectivity.** Default Mode Network (DMN) lateral parietal (LP) ROIs showed negative functional connectivity with Salience (Sal) Network ROIs including supramarginal gyrus (SMG), anterior insula (AIns), and rostral prefrontal cortex (RPFC).

**Fig. 4:**
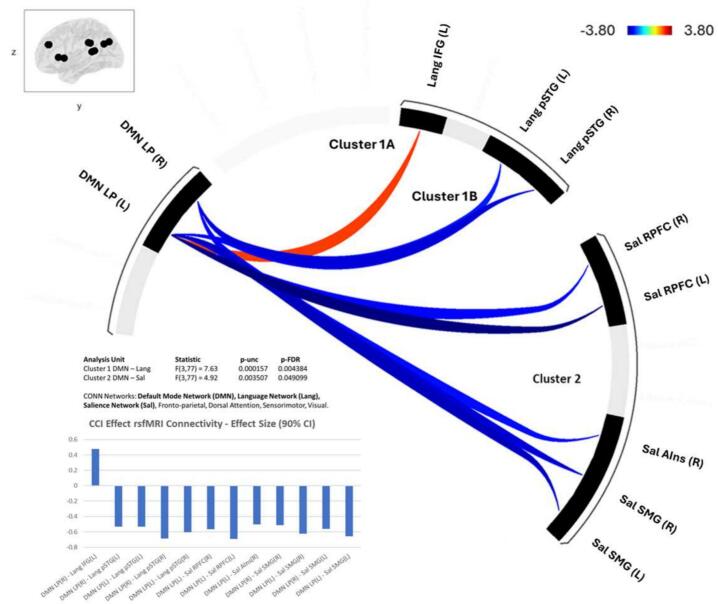
**Default Mode Network – Cognitive Concern Severity Effect on Functional Connectivity**. Default Mode Network (DMN) lateral parietal (LP) ROIs showed functional connectivity associated with cognitive concern severity (CCI score). CCI effects were seen for DMN to Language (Lang) Network ROI positive connectivity with the inferior frontal gyrus (IFG – Cluster 1A) and negative connectivity to superior temporal gyrus (STG – Cluster 1B). CCI effects were also seen for DMN to Salience (Sal) Network ROI negative connectivity with the supramarginal gyrus (SMG), anterior insula (Ains), and rostral prefrontal cortex (RPFC) (Cluster 2).

*Functional Connectivity between DMN and Salience Network*: By contrast, DMN showed negative functional connectivity with Cluster 2 Salience Network regions including rostral prefrontal cortex (rPFC), anterior insula, and supramarginal gyrus (F(3,83) = 66.36, p-corrected < 0.001, Fig. 3). Higher CCI score was associated with higher negative functional connectivity between the DMN left LP region and bilateral rPFC, and right anterior insula, and between DMN bilateral LP regions and bilateral supramarginal gyri (F(3,77) = 4.92, p-corrected < 0.05, Fig. 4).

All other between network ROI to ROI functional connectivity were not significantly associated with CCI score.

### 3.4 SCD-related network functional connectivity and cognitive performance

Between network functional connectivity was calculated as the average connectivity between the identified DMN ROIs and each of the two significantly identified ROI groups: Cluster 1A) Language network-left IFG, Cluster 1B) Language network-bilateral pSTG, Cluster 2) Salience network-bilateral rPFC, right anterior insula, bilateral supramarginal gyri.Post-hoc analysis was conducted for the relationship between functional connectivity strength for each identified significant ROI-ROI pair and cognitive performance measures.

*Cluster 1A: DMN-Language Network IFG Functional Connectivity*.

Cluster 1 A average functional connectivity was associated with Posner task orienting (F(1,72) = 4.10, p = 0.04), with higher positive functional connectivity associated with lower reaction time difference between responding to spatially valid cued targets versus non-cued targets (Table 3). Higher DMN-IFG functional connectivity was associated with lower beneficial effect of orienting cues in visual-spatial attention. There were no other significant associations between Cluster 1A functional connectivity and cognitive performance.

**Table 3:** SCD-related network functional connectivity and cognitive performance.

		**Cognitive Measure**	**df**	**F**	**p**	**β**
**Cluster 1A**	Network Average	Posner – Orienting	72	4.10	0.04	87.76
**Cluster 1B**	Network Average	CRT – Processing RT	76	4.62	0.03	31.88
		Posner – Valid RT	74	4.39	0.04	17.84
	R DMN LP – R Language Pstg	Posner – Valid RT	74	4.53	0.04	73.37
	L DMN LP – R Language pSTG	Posner – Valid RT	74	5.16	0.03	91.12
	R DMN LP – L Language pSTG	CRT – Processing RT	76	5.22	0.03	50.17
	L DMN LP – L Language pSTG	Posner – Valid RT	74	3.97	0.04	72.87
**Cluster 2**	Network Average	CRT – Processing RT	76	8.72	0.004*	61.47
		CRT – Total RT	75	6.58	0.01	92.93
		Posner – Invalid RT	72	10.12	0.002*	69.60
		Posner – Valid RT	74	4.88	0.03	94.30
	L DMN LP – L Salience rPFC	CRT – Processing RT	76	5.27	0.03	44.06
		CRT – Total RT	75	7.54	0.007	126,16
		Posner – Invalid RT	72	14.40	0.003*	107.97
		Posner – Valid RT	74	6.30	0.01	87.36
	L DMN LP – L Salience SMG	CRT – Processing RT	76	7.55	0.01	71.92
		CRT – Total RT	75	5.50	0.02	113.04
		Posner – Valid RT	72	5.83	0.02	91.59
	R DMN LP – R Salience SMG	CRT – Processing RT	76	4.28	0.04	42.06
		CRT – Total RT	75	5.10	0.03	95.19
	L DMN LP – R Salience AIns	CRT – Processing RT	72	4.41	0.04	48.94
LP: lateral parietal, pSTG: posterior superior temporal gyrus, rPFC: rostral prefrontal cortex, SMG: supramarginal gyrus, AIns: anterior insula* Significant following Holm correction for multiple comparisons.						

As Cluster 1A only contained one Language network ROI, exploratory analyses of each ROI to ROI pair was not conducted.

*Cluster 1B: DMN-Language Network pSTG Functional Connectivity*.

Lower Cluster 1B average positive functional connectivity was associated with faster CRT processing RT (F(1,76) = 4.62, p = 0.03) and faster RT for validly cued targets in the Posner task (F(1,74) = 4.39, p = 0.04). There were no other significant associations between Cluster 1B functional connectivity and cognitive performance.

Exploratory analyses of each ROI to ROI pair in Cluster 1B (Table 3) showed that faster RT for validly cued targets in the Posner task was associated with lower right and left DMN LP positive functional connectivity with right and left pSTG. Faster CRT processing RT was associated with lower right DMN LP positive functional connectivity with left pSTG.

*Cluster 2: DMN-Salience Network Functional Connectivity*.

Higher Cluster 2 negative functional connectivity was associated with faster CRT processing RT (F(1,76) = 8.75, p = 0.004) and total RT (F(1,75) = 6.58, p = 0.01) and faster RT in the Posner task for both validly (F(1,74) = 10.12, p = 0.002) and invalidly cued targets (F(1,74) = 4.88, p = 0.03).

Post-hoc analyses of each ROI to ROI pair in Cluster 2 (Table 3) showed that faster CRT processing and total RT was associated with higher negative functional connectivity between: left DMN LP and left Salience rPFC, bilateral DMN LP and left Salience SMG, left DMN LP and right Salience SMG, left DMN LP and right Salience anterior insula (processing RT only). Faster RT for validly and invalidly cues targets in the Posner task was associated with higher negative functional connectivity between left DMN LP and left Salience rPFC, bilateral DMN LP and left Salience SMG (valid cues only), and left DMN LP and left Salience SMG (invalid cues only).

Functional connectivity between ipsilateral DMN LP and Salience SMG, right DMN LP and right Salience SMG, and left DMN LP and right Salience rPFC showed no significant associations with cognitive performance.

## 4 Discussion

This study found that cognitive concern severity (measured by CCI score) in otherwise objectively cognitively unimpaired older adults was associated with slower processing RT. Concern severity was also significantly associated with the strength of functional connectivity between the DMN and Language and Salience networks, and these connectivity patterns were also associated with visual-spatial processing ability measured as psychomotor reaction time.

As the CRT can distinguish motor RT from processing RT, we were able to specifically associate cognitive concern severity with processing RT but not motor RT. The finding that higher CCI score is associated with slower processing speed is consistent with previous work in community-dwelling older adults that found that higher subjective cognitive concerns reported on the Memory Functioning Questionnaire and Everyday Cognition were associated with slower visual processing speed [101]. Significant visual processing speed decline is also a feature of MCI and AD [101], [102]. Slower visual processing speed may interfere with everyday cognitive function by increasing the amount of time needed to process information to be stored in memory and by disturbing the temporal coordination of perceptual and memory processes [37]. These cognitive changes may contribute to the perception of difficulties in cognitive functioning and may represent a form of subtle cognitive decline. There were no other cognitive performance measures that were associated with CCI score, supporting the finding that subjective cognitive concern severity was not associated with objective visual spatial attention or working memory. There were no cognitive measures in which those with lower CCI score performed worse.

Higher CCI score was associated with the functional connectivity strength between DMN, Language Network semantic and visual processing regions [103], and Salience Network regions important for perceptual processing of stimuli and the allocation of internal and external attention [104]. This is noteworthy as the DMN has both direct and indirect roles in cognitive function [105], [106]. Direct functions of the DMN include self-referential perception, personally focused memory, and language and semantic processing. The DMN’s role in language processing includes incorporating new information into existing knowledge and semantic judgments [105], [107], [108], [109].

The current finding of *positive* functional connectivity between the DMN lateral parietal regions and Language Network regions is consistent with the lateral parietal DMN’s role in language comprehension and semantic processing [107], [108], [109]. The identified Language network regions have differential roles in language comprehension that may affect the relationship between DMN functional connectivity and cognitive concern severity. The inferior frontal region is involved in semantic judgement while the superior temporal region is involved in incorporating early visual information into higher order association processes for visual language processing [103], [110]. These regions showed positive functional connectivity with the DMN that decreased with increasing CCI score. For the inferior frontal region, lower DMN functional connectivity was associated with less benefit of visual-spatial cueing on RT. The Language network posterior superior temporal lobe regions also showed positive DMN functional connectivity that declined with higher CCI score; however, reduced positive functional connectivity with DMN was associated with faster visual-spatial processing RT. Changes in connectivity with DMN may have differential effects in these separate regions of the Language network; with only the differences in regions important to visual processing showing a potential compensatory effect on task-related performance. Future work should explore whether DMN-Language Network connectivity patterns are related to language or semantic performance in individuals with significant cognitive concerns.

We also found that higher CCI score was associated with higher *negative* functional connectivity between the DMN and Salience Network. This pattern of higher negative functional connectivity was associated with faster CRT processing RT, and faster RT for all Posner trial types, including invalidly cued trials which require attention shifting. The DMN is involved in internally focused cognitive processing and often shows negative (anti-correlated) functional connectivity with task activated networks [111]. These connectivity patterns reflect the indirect role of DMN deactivation in suppressing internally focused cognitive processes and amplifying task-directed cognition [105], [112], [113], [114], [115]. The Salience Network is involved in detection and processing of external stimuli and the allocation of attention to external/task-focused cognitive processes [104]. The current findings of negative functional connectivity (anti-correlated) between the DMN and the Salience Network reflects the task-off/task-on process of switching between internally focused cognitive processes directed by the DMN and externally focused cognitive processes directed by task-on regions in the Salience Network [112]. Appropriate allocation of cognitive resources and subsequent successful cognitive performance is reliant on the capacity to efficiently engage and disengage the DMN as needed [115]. We hypothesize that stronger coupling between Salience Network activity and DMN deactivation may enhance perceptual processing and attention. Visual processing declines in MCI and AD [102], [116], [117] and may be related to changes within primary visual circuits and the cholinergic system [118]. SCD that results from early AD pathology may also involve subtle visual perceptual decline [101].

DMN functional connectivity with visual processing regions of the Language network and Salience network are related to both higher CCI score and faster visual-spatial processing RT. These patterns may represent compensatory mechanisms that maintain visual spatial attention, where CCI-related functional connectivity patterns were associated with better performance, but there was no association between CCI score and performance. These findings may provide evidence of compensation as the identified connectivity patterns strengthen with greater subjective cognitive concern and may maintain visual spatial attention such that there is no association apparent between objective performance and subjective cognitive concern. For visual processing speed, tighter coupling of task-off/task-on switching between DMN and visual-spatial processes may optimize the allocation of cognitive resources to task-relevant information and counteract primary visual processing decline. However, the remaining association between CCI score and processing speed RT suggests that these mechanisms may not fully counter subtle decline. Self-reported cognitive concerns may represent the subjective experience of these potential compensatory mechanisms as increased required effort or the occasional failure of these mechanisms that results in noticeable cognitive error.

This study has some limitations and should be interpreted with caution. The data are cross-sectional and therefore cannot inform whether the current performance and functional connectivity patterns represent a change over time or with the level of cognitive concern severity within each individual. It may also be possible that participants with subtle objective cognitive decline were included due to the use of MCI/dementia screening assessments that have limited ability to identify subtle impairment. Cross-sectional functional connectivity patterns associated with cognitive concern severity may reflect compensatory changes or deleterious changes related to network inefficiency[119]. While the association with better cognitive performance supports a compensatory interpretation, future work should examine how these patterns change over time and with the progression of dementia pathology and objective cognitive impairment. Examining this hypothesis could help characterize how brain circuits change during the experience of increased self reported cognitive concerns and identify markers for compensatory success or failure.

Additionally, this study included three tasks that assessed visual-spatial processing, attentional orienting and shifting, and working memory. These tasks were chosen to assess primary cognitive domains that are hypothesized to underly more complex cognitive processes and may be involved in cognitive compensation. However, it may be that there are relevant compensatory mechanisms that were not assessed through these tasks. [120]This study did not find an association of functional connectivity with CCI score and working memory performance. Furthermore, due to limited cognitive batteries used in the parent studies, this study did not examine sensitive tasks of episodic memory or language ability, cognitive domains affected in MCI and AD [10], [19], [39]. The post-hoc analysis were not corrected for multiple comparisons following the guidance from Garcia-Perez 2023 [120], however other approaches support correction for individual post-hoc tests. Table 3 indicates which tests would survive Holm correction for multiple comparisons. This correction reduces the number of significant results but maintains the general pattern of results and conclusions.

In conclusion, higher cognitive concern severity (CCI score) in cognitively normal older adults was associated with functional connectivity strength between the DMN and the Language and Salience Networks. The pattern of functional connectivity between the DMN and Language Network was related to both lower beneficial effect of visual-spatial cueing (frontal region) and faster processing reaction time (temporal region), which accords with different roles for Language Network regions in semantic and visual processing.

Functional connectivity between the DMN and Salience Network was anticorrelated (negative) which is consistent with the canonical pattern of activity switching between task-on Salience regions and task-off DMN regions. This negative functional connectivity strength was higher with higher CCI score and was associated with faster processing reaction time. As higher CCI score was associated with slower processing reaction time, while the identified patterns of functional connectivity were generally related to faster processing reaction time, these patterns may reflect compensatory mechanisms that support visual-spatial processing.

This study provides evidence of potential compensatory changes in the functional dynamics between the DMN and brain networks important for allocating attention and visual-spatial processing that support cognitive performance despite increased cognitive concerns. These cognitive processes may provide useful targets for cognitive remediation in aging, as cognitive control [121], [122], [123] and visual-spatial attention[124], [125], [126], [127] have been shown to be amenable to cognitive training and neurofeedback and may have broader effects on cognition including memory. Additionally, pharmacological interventions such as cholinergic enhancement may further support the function of brain networks involved in switching between task-off and task-on states, and the allocation of attention [44], [45], [128], [129], [130]. The development of these approaches or the synergistic combination of cognitive training and pharmacological interventions may improve the perception of cognitive decline in aging and help maintain cognitive performance and functional independence despite neurobiological changes associated with AD.

## Funding

Sources of Financial support: 2R01 AG021476, 4045285625 SAGA-18-418231, 5K01AG073587-02, KL2TR002245, UL1 TR002243, 1S10OD021771-01

## CRediT authorship contribution statement

**Kimberly Albert:** Writing – review & editing, Writing – original draft, Project administration, Methodology, Investigation, Funding acquisition, Formal analysis, Data curation, Conceptualization. **Brian Boyd:** Writing – review & editing, Formal analysis, Data curation. **Brenna McDonald:** Writing – review & editing, Project administration, Investigation, Funding acquisition, Data curation, Conceptualization. **Julie Dumas:** Writing – review & editing, Project administration, Investigation, Funding acquisition, Data curation, Conceptualization. **Andrew Saykin:** Writing – review & editing, Project administration, Methodology, Investigation, Funding acquisition, Data curation, Conceptualization. **Warren Taylor:** Writing – review & editing, Supervision, Project administration, Methodology, Investigation, Funding acquisition, Data curation, Conceptualization. **Paul Newhouse:** Writing – review & editing, Supervision, Resources, Project administration, Methodology, Investigation, Funding acquisition, Data curation, Conceptualization.

## Declaration of competing interest

The authors declare that they have no known competing financial interests or personal relationships that could have appeared to influence the work reported in this paper.

## References

[1] Jessen, F. *et al.* A conceptual framework for research on subjective cognitive decline in preclinical Alzheimer’s disease. *Alzheimer’s and Dementia* vol. 10 844–852 Preprint at 10.1016/j.jalz.2014.01.001+(2014). PMC4317324 24798886

[2] Jessen, F. *et al.* The characterisation of subjective cognitive decline. *The Lancet Neurology* vol. 19 271–278 Preprint at 10.1016/S1474-4422(19)30368-0+(2020). PMC7062546 31958406

[3] Arvanitakis Z. (2018). Memory complaints, dementia, and neuropathology in older blacks and whites. Ann Neurol.

[4] Jessen, F. *et al.* Prediction of Dementia by Subjective Memory Impairment. *Arch Gen Psychiatry* https://doi.org/10.1001/archgenpsychiatry.2010.30 (2010) doi:10.1001/archgenpsychiatry.2010.30. 10.1001/archgenpsychiatry.2010.30 20368517

[5] Mitchell A.J., Beaumont H., Ferguson D., Yadegarfar M., Stubbs B. (2014). Risk of dementia and mild cognitive impairment in older people with subjective memory complaints: meta-analysis. Acta Psychiatr Scand.

[6] Prichep L.S. (2006). Prediction of longitudinal cognitive decline in normal elderly with subjective complaints using electrophysiological imaging. Neurobiol Aging.

[7] Peter J. (2014). Gray matter atrophy pattern in elderly with subjective memory impairment. Alzheimer’s and Dementia.

[8] Van Harten A.C. (2013). Cerebrospinal fluid AB42 is the best predictor of clinical progression in patients with subjective complaints. Alzheimer’s and Dementia.

[9] Wolfsgruber S. (2015). Subjective cognitive decline is related to CSF biomarkers of AD in patients with MCI. Neurology.

[10] Barnes, L. L., Schneider, J. A., Boyle, P. A., Bienias, J. L. & Bennett, D. A. Memory complaints are related to Alzheimer disease pathology in older persons. *Neurology* https://doi.org/10.1212/01.wnl.0000242734.16663.09 (2006) doi:10.1212/01.wnl.0000242734.16663.09. 10.1212/01.wnl.0000242734.16663.09 PMC2740723 17101887

[11] Amariglio R.E. (2012). Subjective cognitive complaints and amyloid burden in cognitively normal older individuals. Neuropsychologia.

[12] Donovan N.J. (2014). Subjective cognitive concerns and neuropsychiatric predictors of progression to the early clinical stages of Alzheimer disease. Am J Geriatr Psychiatr.

[13] Wang, X. *et al.* Neuroimaging advances regarding subjective cognitive decline in preclinical Alzheimer’s disease. *Molecular Neurodegeneration* vol. 15 Preprint at 10.1186/s13024-020-00395-3+(2020). PMC7507636 32962744

[14] Cantero J.L., Iglesias J.E., van Leemput K., Atienza M. (2016). Regional hippocampal atrophy and higher levels of plasma amyloid-beta are associated with subjective memory complaints in nondemented elderly subjects. J Gerontol A Biol Sci Med Sci.

[15] Perrotin A. (2015). Hippocampal subfield volumetry and 3D surface mapping in subjective cognitive decline. J Alzheimer’s Disease.

[16] Meiberth D. (2015). Cortical thinning in individuals with subjective memory impairment. J Alzheimers Dis.

[17] Verfaillie S.C.J. (2016). Thinner temporal and parietal cortex is related to incident clinical progression to dementia in patients with subjective cognitive decline. Alzheimer’s & Dementia : Diagnosis, Assess Disease Monitoring.

[18] Verfaillie S.C.J. (2018). Thinner cortex in patients with subjective cognitive decline is associated with steeper decline of memory. Neurobiol Aging.

[19] McKhann G. (1984). Clinical diagnosis of alzheimer’s disease: Report of the NINCDS-ADRDA work group⋆ under the auspices of department of health and human services task force on alzheimer’s disease. Neurology.

[20] Gauthier S. (2006). Mild cognitive impairment. Lancet.

[21] Ritchie, K. *et al.* Recommended cognitive outcomes in preclinical Alzheimer’s disease: Consensus statement from the European Prevention of Alzheimer’s Dementia project. *Alzheimer’s and Dementia* vol. 13 186–195 Preprint at 10.1016/j.jalz.2016.07.154+(2017). 27702619

[22] Frozza R.L., Lourenco M., De V., Felice F.G. (2018). Challenges for Alzheimer’s disease therapy: Insights from novel mechanisms beyond memory defects. Front Neurosci.

[23] Albert K. (2016). Selective estrogen enhancement of cholinergicrelated cognitive performance in women with or without subjective cognitive decline after menopause. Neuropsychopharmacology.

[24] Albert, K. *et al.* Selective Estrogen Effects On Cholinergic-Related Cognitive Performance And Fmri In Postmenopausal Women With And Without Subjective Cognitive Decline. *Alzheimer’s & Dementia* https://doi.org/10.1016/j.jalz.2017.06.2057 (2017) doi:10.1016/j.jalz.2017.06.2057.

[25] Dumas J.A. (2013). Increased working memory-related brain activity in middle-aged women with cognitive complaints. Neurobiol Aging.

[26] Vega J.N. (2016). Altered brain connectivity in early postmenopausal women with subjective cognitive impairment. Front Neurosci.

[27] Gallardo G., Holtzman D.M. (2019). Amyloid-β and Tau at the Crossroads of Alzheimer’s Disease. Adv Exp Med Biol.

[28] Park D.C., Reuter-Lorenz P. (2009). The adaptive brain: aging and neurocognitive scaffolding. Annu Rev Psychol.

[29] Goh J.O., Park D.C. (2009). Neuroplasticity and cognitive aging: The scaffolding theory of aging and cognition. Restor Neurol Neurosci.

[30] Finke, K., Myers, N., Bublak, P. & Sorg, C. A biased competition account of attention and memory in alzheimer’s disease. *Philosophical Transactions of the Royal Society B: Biological Sciences* vol. 368 Preprint at 10.1098/rstb.2013.0062 (2013). PMC3758205 24018724

[31] Drzezga A. (2011). Neuronal dysfunction and disconnection of cortical hubs in non-demented subjects with elevated amyloid burden. Brain.

[32] Kemppainen N.M. (2007). PET amyloid ligand [11C]PIB uptake is increased in mild cognitive impairment. Neurology.

[33] Insel P.S., Mormino E.C., Aisen P.S., Thompson W.K., Donohue M.C. (2020). Neuroanatomical spread of amyloid β and tau in Alzheimer’s disease: implications for primary prevention. Brain Commun.

[34] Grothe M.J. (2017). In vivo staging of regional amyloid deposition. Neurology.

[35] Chun, M. M. & Turk-Browne, N. B. Interactions between attention and memory. *Current Opinion in Neurobiology* vol. 17 177–184 Preprint at 10.1016/j.conb.2007.03.005 (2007). 17379501

[36] Aly, M. & Turk-Browne, N. B. Attention Stabilizes Representations in the Human Hippocampus. *Cerebral Cortex* https://doi.org/10.1093/cercor/bhv041 (2016) doi:10.1093/cercor/bhv041. 10.1093/cercor/bhv041 PMC4712804 25766839

[37] Uncapher, M. R. & Rugg, M. D. Selecting for memory? The influence of selective attention on the mnemonic binding of contextual information. *Journal of Neuroscience* https://doi.org/10.1523/JNEUROSCI.1043-09.2009 (2009) doi:10.1523/JNEUROSCI.1043-09.2009. 10.1523/JNEUROSCI.1043-09.2009 PMC2730727 19553466

[38] Grady C., Sarraf S., Saverino C., Campbell K. (2016). Age differences in the functional interactions among the default, frontoparietal control, and dorsal attention networks. Neurobiol Aging.

[39] Carter, S. F., Caine, D., Burns, A., Herholz, K. & Ralph, M. A. L. Staging of the cognitive decline in Alzheimer’s disease: Insights from a detailed neuropsychological investigation of mild cognitive impairment and mild Alzheimer’s disease. *Int J Geriatr Psychiatry* https://doi.org/10.1002/gps.2738 (2012) doi:10.1002/gps.2738. 10.1002/gps.2738 21618285

[40] McDonough I.M., Wood M.M., Miller W.S. (2019). A review on the trajectory of attentional mechanisms in aging and the alzheimer’s disease continuum through the attention network test. Yale J Biology Med Preprint at.

[41] Salthouse T.A. (1996). The processing-speed theory of adult age differences in cognition. Psychol Rev.

[42] Somberg B.L., Salthouse T.A. (1982). Divided attention abilities in young and old adults. J Exp Psychol Hum Percept Perform.

[43] Dumas, J. A. & Newhouse, P. A. The cholinergic hypothesis of cognitive aging revisited again: Cholinergic functional compensation. *Pharmacology Biochemistry and Behavior* vol. 99 254–261 Preprint at 10.1016/j.pbb.2011.02.022 (2011). PMC3114182 21382398

[44] Sarter M., Paolone G. (2011). Deficits in attentional control: Cholinergic mechanisms and circuitry-based treatment approaches. Behav Neurosci.

[45] Parikh V., Sarter M. (2008). Cholinergic mediation of attention: Contributions of phasic and tonic increases in prefrontal cholinergic activity. Ann N Y Acad Sci.

[46] Wang X. (2020). Neuroimaging advances regarding subjective cognitive decline in preclinical Alzheimer’s disease. Mol Neurodegener.

[47] Schultz S.A. (2015). Subjective memory complaints, cortical thinning, and cognitive dysfunction in middle-aged adults at risk for AD. Alzheimers Dement (Amst).

[48] Scheef L. (2019). Subregional volume reduction of the cholinergic forebrain in subjective cognitive decline (SCD). Neuroimage Clin.

[49] Shokouhi S. (2020). CHOLINERGIC BASAL FOREBRAIN VOLUME CHANGES IN COGNITIVELY NORMAL ELDERLY ADULTS AND PRECLINICAL ALZHEIMER’S DISEASE. Am J Geriatr Psychiatry.

[50] Shokouhi S. (2019). The relationship between domain-specific subjective cognitive decline and Alzheimer’s pathology in normal elderly adults. Neurobiol Aging.

[51] Rodriguez-Hernandez M.A. (2024). Degeneration of the cholinergic system in individuals with subjective cognitive decline: A systematic review. Neurosci Biobehav Rev.

[52] Viviano, R. P. & Damoiseaux, J. S. Functional neuroimaging in subjective cognitive decline: Current status and a research path forward. *Alzheimer’s Research and Therapy* vol. 12 Preprint at 10.1186/s13195-020-00591-9 (2020). PMC7063727 32151277

[53] Rodda J.E., Dannhauser T.M., Cutinha D.J., Shergill S.S., Walker Z. (2009). Subjective cognitive impairment: increased prefrontal cortex activation compared to controls during an encoding task. Int J Geriatr Psychiatry.

[54] Erk S. (2011). Evidence of neuronal compensation during episodic memory in subjective memory impairment. Arch Gen Psychiatry.

[55] Fastame M.C. (2022). Are subjective cognitive complaints associated with executive functions and mental health of older adults?. Cogn Process.

[56] Hohman T.J., Beason-Held L.L., Lamar M., Resnick S.M. (2011). Subjective cognitive complaints and longitudinal changes in memory and brain function. Neuropsychology.

[57] Schultz A.P. (2017). Phases of hyperconnectivity and hypoconnectivity in the default mode and salience networks track with amyloid and tau in clinically normal individuals. J Neurosci.

[58] Roemer-Cassiano S.N. (2025). Amyloid-associated hyperconnectivity drives tau spread across connected brain regions in Alzheimer’s disease. Sci Transl Med.

[59] Penalba-Sánchez L., Oliveira-Silva P., Sumich A.L., Cifre I. (2023). Increased functional connectivity patterns in mild Alzheimer’s disease: A rsfMRI study. Front Aging Neurosci.

[60] Hayes J.M. (2017). Subjective memory complaints are associated with brain activation supporting successful memory encoding. Neurobiol Aging.

[61] Dong C. (2018). Altered functional connectivity strength in informant-reported subjective cognitive decline: A resting-state functional magnetic resonance imaging study. Alzheimer’s & Dementia: Diagnosis, Assess Disease Monitoring.

[62] Viviano R.P. (2018). Aberrant memory system connectivity and working memory performance in subjective cognitive decline. NeuroImage Preprint at.

[63] Dillen K.N.H. (2016). Aberrant functional connectivity differentiates retrosplenial cortex from posterior cingulate cortex in prodromal Alzheimer’s disease. Neurobiol Aging.

[64] Saykin A.J. (2006). Older adults with cognitive complaints show brain atrophy similar to that of amnestic MCI. Neurology.

[65] Hindmarch I. (1984). Psychological performance models as indicators of the effects of hypnotic drugs on sleep. Psychopharmacology Suppl.

[66] Posner M.I. (1980). Posner - 1980 - Orienting of attention. J Exp Psychol.

[67] Dumas J.A. (2008). Nicotinic versus muscarinic blockade alters verbal working memory-related brain activity in older women. Am J Geriatr Psychiatry.

[68] Folstein, M. F., Robins, L. N. & Helzer, J. E. The Mini-Mental State Examination. *Archives of General Psychiatry* vol. 40 Preprint at 10.1001/archpsyc.1983.01790060110016 (1983). 6860082

[69] Nasreddine Z.S. (2005). The montreal cognitive assessment, MoCA: A brief screening tool for mild cognitive impairment. J Am Geriatr Soc.

[70] Rescorla L.A., Achenbach T.M. (2004). *The use of psychological testing for treatment planning and outcomes assessment: Volume 3: Instruments for adults*.

[71] Reisberg B., Ferris S.H. (1988). Brief cognitive rating scale (BCRS). Psychopharmacol Bull.

[72] Reisberg B., Ferris S.H., De Leon M.J., Crook T. (1982). The global deterioration scale for assessment of primary degenerative dementia. Am J Psychiatry.

[73] Rabin L.A. (2015). Subjective cognitive decline in older adults: An overview of self-report measures used across 19 international research studies. J Alzheimer’s Disease.

[74] Newhouse P.A. (2020). Cognitive symptoms in early postmenopausal women: Relationship to brain structure. Alzheimer’s & Dementia.

[75] Molinuevo, J. L. *et al.* Implementation of subjective cognitive decline criteria in research studies. *Alzheimer’s and Dementia* vol. 13 296–311 Preprint at 10.1016/j.jalz.2016.09.012 (2017). PMC5344703 27825022

[76] Engle, R. W. & Kane, M. J. Executive Attention, Working Memory Capacity, and a Two-Factor Theory of Cognitive Control. *The psychology of learning and motivation: Advances in research and theory, Vol. 44.* 145–199 Preprint at 10.1016/S0079-7421(03)44005-X (2004).

[77] Cowan, N. *et al.* On the capacity of attention: Its estimation and its role in working memory and cognitive aptitudes. *Cognitive Psychology* vol. 51 42–100 Preprint at 10.1016/j.cogpsych.2004.12.001 (2005). PMC2673732 16039935

[78] Majerus S. (2012). Attention supports verbal short-term memory via competition between dorsal and ventral attention networks. Cereb Cortex.

[79] Kirchner W.K. (1958). Age differences in short-term retention of rapidly changing information. J Exp Psychol.

[80] Whitfield-Gabrieli S., Nieto-Castanon A. (2012). Conn: A functional connectivity toolbox for correlated and anticorrelated brain networks. Brain Connect.

[81] Nieto-Castanon, A. & Whitfield-Gabrieli, S. *CONN Functional Connectivity Toolbox: RRID* SCR_009550, *Release 21*. *CONN functional connectivity toolbox: RRID* SCR_009550, *release 21* (2021). doi:10.56441/hilbertpress.2161.7292. 10.1089/brain.2012.0073 22642651

[82] Flandin G., Friston K. (2008). Statistical parametric mapping (SPM). Scholarpedia.

[83] Glasser M.F. (2013). The minimal preprocessing pipelines for the Human Connectome Project. Neuroimage.

[84] Andersson J.L.R., Hutton C., Ashburner J., Turner R., Friston K. (2001). Modeling geometric deformations in EPI time series. Neuroimage.

[85] Friston K.J. (1995). Spatial registration and normalization of images. Hum Brain Mapp.

[86] Henson R., Büchel C., Josephs O., Fristen K. (1999). The slice-timing problem in event-related fMRI. Neuroimage.

[87] Sladky R. (2011). Slice-timing effects and their correction in functional MRI. Neuroimage.

[88] Power J.D. (2014). Methods to detect, characterize, and remove motion artifact in resting state fMRI. Neuroimage.

[89] Ashburner J. (2016). Preparing fMRI data for statistical analysis. NeuroMethods.

[90] Calhoun V.D. (2017). The impact of T1 versus EPI spatial normalization templates for fMRI data analyses. Hum Brain Mapp.

[91] Ashburner J., Friston K.J. (2005). Unified segmentation. Neuroimage.

[92] Ashburner J. (2007). A fast diffeomorphic image registration algorithm. Neuroimage.

[93] Nieto-Castanon A. (2020). FMRI denoising pipeline. Handbook of functional connectivity Magnetic Resonance Imaging methods in CONN.

[94] Friston K.J., Williams S., Howard R., Frackowiak R.S.J., Turner R. (1996). Movement-related effects in fMRI time-series. Magn Reson Med.

[95] Hallquist M.N., Hwang K., Luna B. (2013). The nuisance of nuisance regression: Spectral misspecification in a common approach to resting-state fMRI preprocessing reintroduces noise and obscures functional connectivity. Neuroimage.

[96] Behzadi Y., Restom K., Liau J., Liu T.T. (2007). A component based noise correction method (CompCor) for BOLD and perfusion based fMRI. Neuroimage.

[97] Chai X.J., Castañán A.N., Öngür D., Whitfield-Gabrieli S. (2012). Anticorrelations in resting state networks without global signal regression. Neuroimage.

[98] Nieto-Castanon A. (2020). Functional Connectivity measures. Handbook of functional connectivity Magnetic Resonance Imaging methods in CONN.

[99] Nieto-Castanon A. (2020). General Linear Model. Handbook of functional connectivity Magnetic Resonance Imaging methods in CONN.

[100] Chumbley J., Worsley K., Flandin G., Friston K. (2010). Topological FDR for neuroimaging. Neuroimage.

[101] Marrero-Polegre D. (2023). Lower visual processing speed relates to greater subjective cognitive complaints in community-dwelling healthy older adults. Front Psych.

[102] Ruiz-Rizzo A.L. (2017). Simultaneous object perception deficits are related to reduced visual processing speed in amnestic mild cognitive impairment. Neurobiol Aging.

[103] Rolls E.T., Deco G., Huang C.C., Feng J. (2022). The human language effective connectome. Neuroimage.

[104] Seeley W.W. (2007). Dissociable intrinsic connectivity networks for salience processing and executive control. J Neurosci.

[105] Menon, V. 20 years of the default mode network: A review and synthesis. *Neuron* vol. 111 Preprint at https://doi.org/10.1016/j.neuron.2023.04.023 (2023). 10.1016/j.neuron.2023.04.023 PMC10524518 37167968

[106] Mancuso L. (2022). Tasks activating the default mode network map multiple functional systems. Brain Struct Funct.

[107] Binder, J. R. & Desai, R. H. The neurobiology of semantic memory. *Trends in Cognitive Sciences* vol. 15 Preprint at 10.1016/j.tics.2011.10.001 (2011). PMC3350748 22001867

[108] Humphries C., Binder J.R., Medler D.A., Liebenthal E. (2007). Time course of semantic processes during sentence comprehension: An fMRI study. Neuroimage.

[109] Binder J.R., Desai R.H., Graves W.W., Conant L.L. (2009). Where is the semantic system? A critical review and meta-analysis of 120 functional neuroimaging studies. Cereb Cortex.

[110] Garcia A., Cohen R.A., Porges E.C., Williamson J.B., Woods A.J. (2022). Functional connectivity of brain networks during semantic processing in older adults. Front Aging Neurosci.

[111] Fox M.D. (2005). The human brain is intrinsically organized into dynamic, anticorrelated functional networks. PNAS.

[112] Raichle M.E. (2015). The brain’s default mode network. Annu Rev Neurosci.

[113] Sheline Y.I. (2009). The default mode network and self-referential processes in depression. PNAS.

[114] Horn A., Ostwald D., Reisert M., Blankenburg F. (2014). The structural-functional connectome and the default mode network of the human brain. Neuroimage.

[115] Mohan A. (2016). The significance of the Default Mode Network (DMN) in neurological and neuropsychiatric disorders: A review. Yale J Biology Med Preprint at.

[116] Kavcic V., Vaughn W., Duffy C.J. (2011). Distinct visual motion processing impairments in aging and Alzheimer’s disease. Vision Res.

[117] Haupt M. (2021). Phasic alerting increases visual processing speed in amnestic mild cognitive impairment. Neurobiol Aging.

[118] Kusne, Y., Wolf, A. B., Townley, K., Conway, M. & Peyman, G. A. Visual system manifestations of Alzheimer’s disease. *Acta Ophthalmologica* vol. 95 Preprint at 10.1111/aos.13319 (2017). 27864881

[119] Hillary F.G., Grafman J.H. (2017). Injured Brains and Adaptive Networks: the benefits and costs of hyperconnectivity. Trends Cogn Sci.

[120] García-Pérez M.A. (2023). Use and misuse of corrections for multiple testing. Methods in Psychology.

[121] Morimoto S.S. (2020). Targeting cognitive control deficits with neuroplasticity-based computerized cognitive remediation in patients with geriatric major depression: A randomized, double-blind, controlled trial. Am J Geriatr Psychiatr.

[122] Newhouse P., Morimoto S., Vega J., Manning K., Conley A. (2024). Digital therapeutics for geriatric psychiatry: From laboratory to clinic. Am J Geriatr Psychiatry.

[123] Vega J.N. (2023). Use of focused computerized cognitive training (Neuroflex) to improve symptoms in women with persistent chemotherapy-related cognitive impairment. Digit Health.

[124] Jiang Y., Abiri R., Zhao X. (2017). Tuning up the old brain with new tricks: Attention training via neurofeedback. Front Aging Neurosci.

[125] Tang Y.Y., Tang R., Posner M.I., Gross J.J. (2022). Effortless training of attention and self-control: mechanisms and applications. Trends Cogn Sci.

[126] Kim K., Lee J.H. (2022). The effect of feedback in virtual attention training on orienting attention in individuals with sluggish cognitive tempo. J Atten Disord.

[127] Anguera J.A. (2022). *npj aging***8**.

[128] Thiel C.M., Fink G.R. (2008). Effects of the cholinergic agonist nicotine on reorienting of visual spatial attention and top-down attentional control. Neuroscience.

[129] Bentley P., Vuilleumier P., Thiel C.M., Driver J., Dolan R.J. (2003). Cholinergic enhancement modulates neural correlates of selective attention and emotional processing. Neuroimage.

[130] Furey M.L., Pietrini P., Haxby J.V., Drevets W.C. (2008). Selective effects of cholinergic modulation on task performance during selective attention. Neuropsychopharmacology.

